# Outcome of 881 total hip arthroplasties in 747 patients 21 years or younger: data from the Nordic Arthroplasty Register Association (NARA) 1995–2016

**DOI:** 10.1080/17453674.2019.1615263

**Published:** 2019-05-15

**Authors:** Vera Halvorsen, Anne Marie Fenstad, Lars B Engesæter, Lars Nordsletten, Søren Overgaard, Alma B Pedersen, Johan Kärrholm, Maziar Mohaddes, Antti Eskelinen, Keijo T Mäkelä, Stephan M Röhrl

**Affiliations:** a Division of Orthopedic Surgery, Oslo University Hospital Ulleval, Oslo, Norway;; b Norwegian Arthroplasty Registry, Department of Orthopaedic Surgery, Haukeland University Hospital, Bergen, Norway;; c Department of Clinical Medicine, University of Bergen, Bergen, Norway;; d University of Oslo, Oslo, Norway;; e Department of Orthopaedic Surgery, Traumatology and Odense University Hospital, and Department of Clinical Research, University of Southern Denmark, Odense, Denmark;; f Danish Hip Arthroplasty Register, Denmark;; g Department of Clinical Epidemiology, Aarhus University Hospital, Aarhus, Denmark;; h The Swedish Hip Arthroplasty Register, Sweden;; i Department of Orthopaedics, Institute of Surgical Sciences, Sahlgrenska University Hospital, University of Gothenburg, Sweden;; j Coxa Hospital for Joint Replacement, and Faculty of Medicine and Life Sciences, University of Tampere, Tampere, Finland;; k The Finnish Arthroplasty Register, Finland;; l Department of Orthopaedics and Traumatology, Turku University Hospital, Turku.

## Abstract

Background and purpose — The literature is scarce on the outcome of the youngest patients with total hip arthroplasties (THAs). We analyzed register data, revision risk, and related factors in patients 21 years or younger with THAs in the Nordic Arthroplasty Register Association (NARA).

Patients and methods — We included all THA patients 21 years or younger reported during 1995 through 2016 to the Danish, Finnish, Norwegian, and Swedish hip arthroplasty registers and merged these into the NARA dataset. Primary outcome was any implant revision.

Results — We identified 881 THAs in 747 patients. Mean age at primary surgery was 18 years (9–21). The indications for THA were pediatric hip diseases (33%), systemic inflammatory disease (23%), osteoarthritis (4%), avascular necrosis (12%), hip fracture sequelae (7%), and other diagnoses (21%). Unadjusted 10-year survival for all THAs was 86%. Comparison between indications showed no differences in survival. Uncemented implants were used most frequently. Survival for uncemented and cemented implants was the same adjusted for sex, indication, head size, and time period for primary surgery. Aseptic loosening was the main cause of revision.

Interpretation — Both cemented and uncemented fixations seem to be a viable option in this age group, but with a lower implant survival than in older patient groups.

In children and adolescents small size of the hip bones and anatomic changes due to underlying disease may make total hip arthroplasty (THA) technically demanding, and in the youngest high activity level is a risk factor for revision (Munger et al. 2006, Flugsrud et al. 2007, Prokopetz et al. 2012). It is reasonable to expect revision surgery throughout the patient’s lifespan. In all ages the reduced bone stock after revision surgery may cause later problems, the implant survival may be poor and recovery of function is more strenuous (Lie et al. 2004, Bischel et al. 2012, Adelani et al. 2014, Goodman et al. 2014). Reports decades ago on THAs in patients younger than 21 years were mostly on patients with juvenile chronic arthropathies and most of the components were cemented (Ruddlesdin et al. 1986, Witt et al. 1991, Cage et al. 1992). Historically, long-time cohorts of cemented THAs demonstrate down to 50% implant survival after 12–19 years (Torchia et al. 1996, Wroblewski et al. 2010). Later studies have found more promising results with uncemented implants. Hannouche et al. (2016) found an estimated 90% survival after 10 years in patients aged less than 20 years with almost exclusively uncemented implants with ceramic-on-ceramic bearings. Tsukanaka et al. (2016) found a 10-year survival rate of 70% in a Norwegian register study of 96 cemented and uncemented hips in 81 patients aged less than 20 years. A recent register study of 769 THAs in patients 20 years or younger from England, Wales, Northern Ireland, and the Isle of Man reported a 5-year implant survival of 96%. The patients included were from the period 2003–2017 (Metcalfe et al. 2018).

We present an epidemiologic overview on more recent primary THAs regarding indications and fixation concepts. Implant survivorship and reasons for revision were evaluated. Patients operated in the first years of the study period may have limited applicability to contemporary THAs because of older implant design. Our hypothesis was that newer implants might have better survival compared with those used in the earlier cohort.

## Patients and methods

### Data source

This study was based on data from the Nordic Arthroplasty Register Association (NARA), which is a collaboration between the national joint replacement registers in Denmark, Finland, Norway, and Sweden (Mäkelä et al. 2014). Data from the registers have been merged into a common NARA minimal dataset combining available parameters in all four countries. The study is reported according to RECORD guidelines.

### Study population

Using the NARA database, we identified all patients 21 years or younger (n = 747) reported to have had 881 primary THAs during 1995–2016.

### Covariates

**Figure 1.**
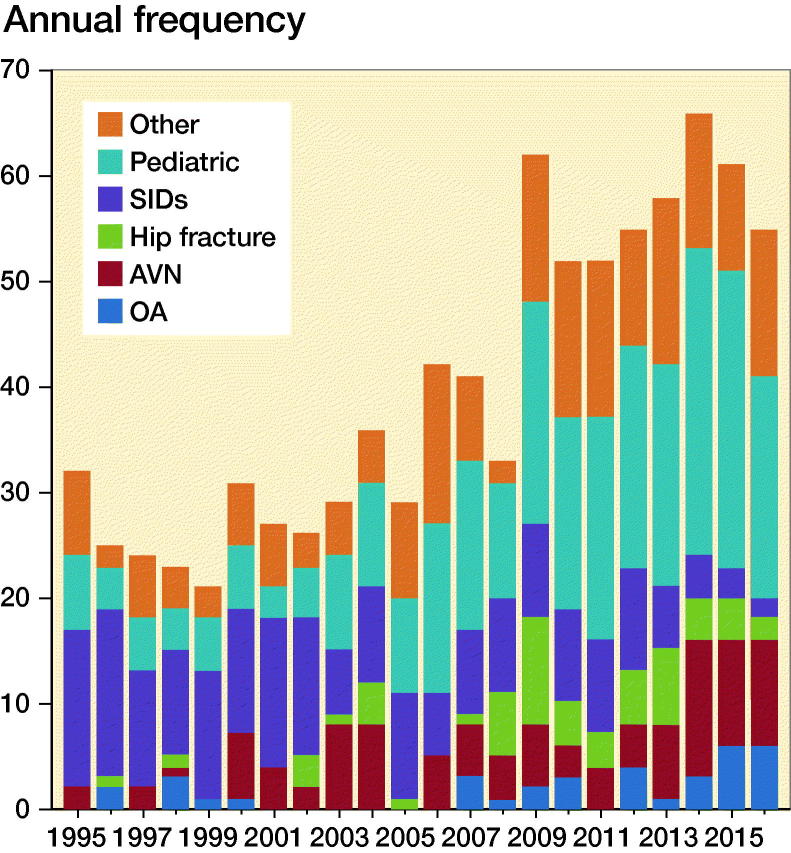
Indications for THAs in patients 21 years or younger in NARA countries 1995–2016. Other: tumors, sequelae after infection, pharmaceutically induced femoral necrosis. Pediatric: developmental dysplasia of the hip (DDH), Perthes, slipped capital femoral epiphysis (SCFE). SIDs: systemic inflammatory diseases including rheumatoid arthritis, ankylosing spondylitis, and other inflammatory diseases. AVN: avascular necrosis. OA: osteoarthritis.

Information on country of origin, age, sex, indication for THA, calendar year of surgery, type of fixation, implants and articulations, approach, cause of any revision, and date of death were collected. No THAs were excluded even if there were missing values in some categories in the dataset. The diagnoses for primary THA were grouped into 6 categories (Figure 1).

### Outcome

The primary outcome measure was time to 1st revision. A revision was defined as any removal or exchange of components. Revisions were categorized as change of cup, change of stem, change of both stem and cup, or removal of components. In the NARA minimal dataset changing of liner is recorded as cup revision. Only the 1st revision was reported.

### Statistics

Categorical data were cross-tabulated by the chi-square test. Continuous data were described using means (SD), and possible differences were tested with Student’s t-test. All tests were 2-sided and the significance level was set to 0.05. The Kaplan–Meier method was used to calculate unadjusted survival functions and estimates with 95% confidence interval (CI). In order to examine the association between sex, diagnosis, type of fixation, femoral head size, calendar period, and risk of revision, we calculated the hazard ratio (HR) using Cox regression analyses, crude HR, and HR mutually adjusted for all covariates. We used log–log plots and Schoenfeld residuals for each covariate to test that the Cox proportional hazard model was fulfilled. Bilateral observations do not introduce significant dependency problems in register studies, hence these were included (Ranstam et al. 2011).

### Ethics, funding, and potential conflict of interest

Ethical approval: Denmark: Danish Data Protection Agency nr. 1-16-02-54-17, Finland: National Institute of Helath and Welfare (THL): Dnro THL/1743/5.05.00/2014, Norway: Norwegian Data Protection Authority 03/00058-20/CGN, Sweden: DNR 804-17 Regional ethical committee Gothenburg. Research was funded by NordForsk Grant for NARA.No conflict of interest was reported by the authors.

## Results

### Demographic data and diagnosis

The number of primary THAs in patients 21 years or younger was 881 (0.1%) compared with 745,827 primary THAs in patients of all ages during the 22-year study period. Of the 881 THAs, 134 were bilateral procedures (18%). 43% of the bilateral cases were performed in systemic inflammatory disease patients (SID patients).

The male:female ratio was close to 1:1, except in Sweden where the ratio was almost 1:2. Mean age at primary THA was 18 (9–21) years (SD 2.4). Mean age at primary surgery was similar for males and females. Pediatric hip disease was the most common indication for THA accounting for 33% of the patients; the second largest group was SID with 23%, OA accounted for 4%, AVN for 12%, hip fractures for 7%, and other for 21%. The indications for THA varied during the study period; there was a trend towards declining frequency of SID and increasing frequency of pediatric diseases (Figure 1, Table 1). There were differences among the Nordic countries between indications, particularly the pediatric and SID groups, which varied from 11% to 51% and 17% to 35% respectively (Table 1).

**Table 1.** Patient demographics for each country with a total of 881 primary THAs in 1995–2016 (numbers in parentheses)

Country	Denmark	Finland	Norway	Sweden	Total
THAs per country, n (%)	253.(29)	171.(19)	207.(24)	250.(28)	881
Revisions, n (%)	28.(11)	30.(18)	17.(8)	43.(17)	118.(13)
Number of deaths	6	2	7	8	23
Sex, % male	53	52	50	36	47
Age, mean (SD)	17.9 (2.3)	18.3 (2.4)	17.8 (2.4)	18.1 (2.4)	18.0 (2.4)
Indications, %					
Osteoarthritis	2.4	8.8	1.4	4.8	4.1
Avascular necrosis	11	15	11	12	12
Hip fracture	5.6	7.6	5.3	7.6	6.5
SID^a^	18	21	17	35	23
Pediatric	48	11	51	18	33
Other	15	36	15	23	22

In 1.2% of the operations information on indication was missing.

a Systemic inflammatory disease.

### Fixations, head size, articulations, implants, and surgical approach

In total, 74% of the implants were uncemented (Table 2). Hip resurfacing arthroplasty was rare (3.6%). We found no obvious association between diagnosis and type of fixation, which was equally distributed among different diagnoses. 25% of the heads were 32 mm and 46% were smaller. Metal on highly crosslinked polyethylene was the most frequent articulation (18%). Type of articulation was missing in 19% of cases. A posterior approach was used in 47% of the operations (Table 2).

**Table 2.** Fixations, head sizes, articulations, surgical approach, and trochanteric osteotomies (n = 881). Values are frequency (%)

Fixations	
Cemented	62 (7.0)
Uncemented	659 (74)
Hybrid	36 (4.1)
Reverse hybrid	78 (8.9)
Resurfacing	31 (3.5)
Missing	15 (1.7)
Head size	
< 32 mm	405 (46)
32 mm	221 (25)
> 32 mm	180 (20)
Missing	75 (8.5)
Articulation	
Metal/metal	145 (17)
Metal/ceramic	1 (0.1)
Ceramic/ceramic	97 (11)
PolyXL/metal	206 (23)
PolyXL/ceramic	135 (15)
Poly/metal	78 (8.9)
Poly/ceramic	54 (6.1)
Missing	165 (19)
Posterior approach^a^	
Yes	418 (47)
No	262 (30)
Missing	201 (23)
Trochanteric osteotomy	
Yes	21 (2.4)
No	658 (75)
Missing^a^	202 (23)

a Finland did not report approach.

### Implant brands

The number of different brands varied from 9 to 22 for cups and 10 to 21 for stems for each of the participating countries. The variety of brands was similar in the latter period (2012–2016) compared with the first two (1995–2004 and 2005–2011). Implant survival estimates linked to brands cannot be performed due to the heterogenicity of the material and the relatively small number of THAs.

### Implant survival and revisions

**Figure 2.**
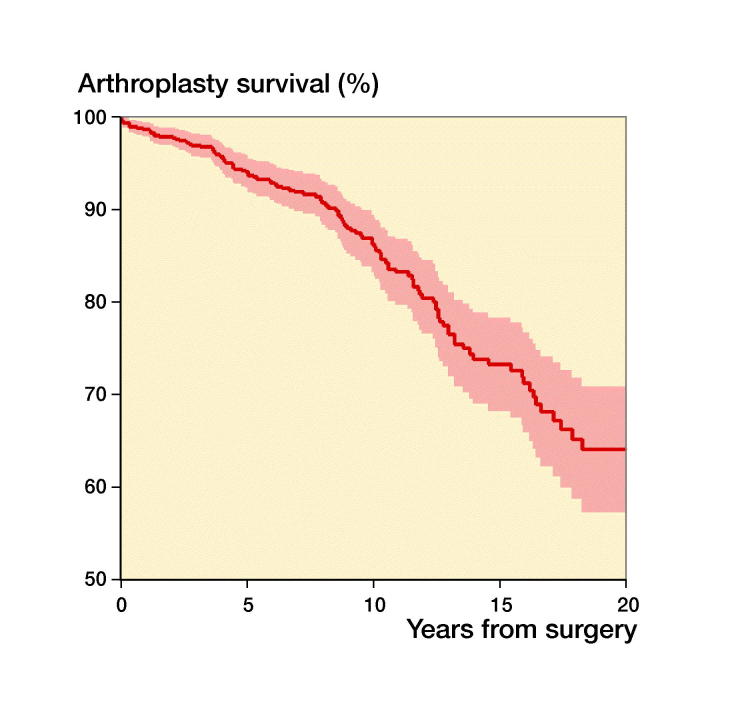
Kaplan–Meier revisionfree survival curve. Confidence intervals are shaded. The 10-year survival was 86%.

**Figure 3.**
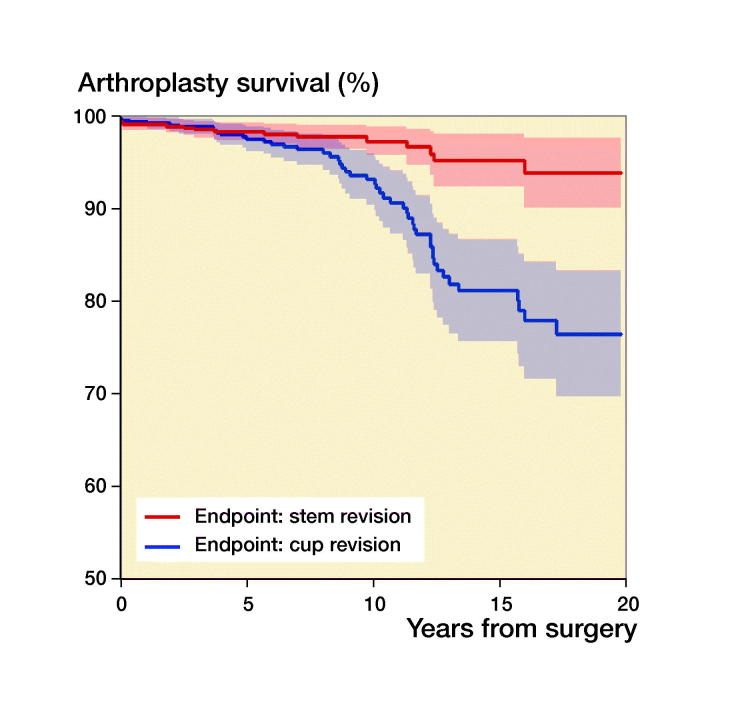
Kaplan–Meier survival curves for cups (red) and stems (blue). Confidence interval are shaded. Data from Finland are not included because surgical procedure at revision is not registered.

118 (13%) of the 881 THAs were revised during the study period (Table 1). With any reason for revision as endpoint, the 5-year unadjusted survival was 94% (CI 92–96), the 10-year survival was 86% (CI 83–89), and the 15-year survival 73% (CI 68–78) (Figure 2). Cups had a higher revision rate than stems (Figure 3). There were 4 types of fixations: cemented, uncemented, hybrid (cup uncemented, stem cemented), and reverse hybrid (cup cemented, stem uncemented) and in addition 31 resurfacing arthroplasties (3.6%). Adjusted data showed no statistically significant survival difference between cemented and uncemented implants (p = 0.2, Table 4).

The numbers per disease were too small to make meaningful estimates of survivorship related to disease.

Hazard ratio (HR) for revision was analyzed in different time periods (Table 4). Unadjusted HR was 0.5 (CI 0.3–0.8) for the years 2005–2011 and 0.4 (CI 0.1–1.0) for 2012–2016 compared with 1995–2004. Adjusted for sex, indication, fixation, and head size the HR in the period 2005–2011 was 0.5 (CI 0.3–0.9) and 0.6 (CI 0.2–1.8) in the period 2012–2016 compared with 1995–2004. In 2012–2016 only 5 revisions were recorded. Using the period 1995–2011 as HR reference, HR in the period 2012–2016 was 0.5 (CI 0.2–1.3) (data not shown in table). The hazard ratio for revision was 2.5 (95% CI 1.4–4.5) higher for reverse hybrid than for uncemented fixation (Table 4).

There were no revisions recorded where removal of components took place. That would probably have been the case if more than the first revision had been reported. Most of the revisions, 52%, were due to aseptic loosening (Table 3).

**Table 3.** Causes of revisions and revision procedures performed (n = 118). Values are frequency (%)

Cause	
Aseptic loosening	61 (52)
Deep infection	6 (5.1)
Periprosthetic fracture	3 (2.5)
Dislocation	11 (9.3)
Pain only	1 (0.8)
Other	36 (31)
Procedure^a^	
Total replaced	11 (9.3)
Only stem replaced	6 (5.1)
Only cup or liner replaced	39 (33)
Girdlestone	3 (2.5)
Other	29 (25)
Missing	30 (25)

a Finland did not report procedure. Denmark did not differ between cup, stem, or total revision and data are reported as “other.”

**Table 4.** Patient- and procedure-related risk factors for THA revision in patients 21 years and younger at surgery adjusted for sex, indications, head size, implant fixation, and time period (n = 881)

Variables	Total number	No. of revisions	Unadjusted HR (95% CI)	p-value	Adjusted HR (95%CI)	p-value
Sex						
Men	418	46	1		1	
Women	463	72	1.1 (0.8–1.6)	0.6	1.1 (0.7–1.7)	0.8
Indication (n = 879):						
Pediatric	290	30	1		1	
Osteoarthritis	36	1	0.2 (0.1–1.7)	0.2	–	
Avascular necrosis	104	7	0.6 (0.2–1.3)	0.2	0.5 (0.2–1.3)	0.2
Hip fracture	57	6	1.2 (0.5–2.8)	0.7	1.5 (0.5–3.9)	0.5
SIDs	203	52	1.2 (0.8–1.9)	0.4	0.9 (0.5–1.7)	0.9
Other	189	22	1.0 (0.5–1.7)	0.9	1.1 (0.6–2.1)	0.7
Fixation (n = 866):						
Uncemented	659	69	1		1	
Cemented	62	20	1.9 (1.2–3.2)	0.01	1.6 (0.8–3.2)	0.2
Hybrid	36	5	0.7 (0.3–1.8)	0.5	0.5 (0.2–1.8)	0.3
Reverse hybrid	78	17	2.4 (1.4–4.0)	0.002	2.5 (1.4–4.5)	0.002
Resurfacing	31	7	1.8 (0.8–4.0)	0.1	0.9 (0.2–4.7)	0.9
Head size (n = 806):						
< 32 mm	405	76	1		1	
32 mm	221	4	0.3 (0.1–0.8)	0.01	0.4 (0.1–1.3)	0.1
> 32 mm	180	8	0.4 (0.2–0.8)	0.02	0.7 (0.3–1.9)	0.5
Time period:						
1995–2004	274	90	1		1	
2005–2011	311	23	0.5 (0.3–0.8)	0.007	0.5 (0.3–0.9)	0.02
2012–2016	296	5	0.4 (0.1–1.0)	0.05	0.6 (0.2–1.8)	0.4

## Discussion

The overall 10-year implant survival was 86%. There was no difference in adjusted survival for cemented and uncemented implants. Reverse hybrid fixation had a higher hazard ratio for revision than other fixations. We could not show a convincing trend towards better survivorship in the latter time period.

One-third of the patients had pediatric hip disease. The increase in the number of pediatric hip disease indications cannot be explained by changes in DDH screening since the screening programs have been unchanged over the years. Neither have any changes in the incidences of Perthes disease or slipped capital femoral epiphysis been reported in the literature. There are also differences between the countries concerning pediatric hip disease as indication, but a study of hip radiograms at skeletal maturity showed that the prevalence of developmental dysplasia of the hip is on the same level in the Nordic countries (Engesaeter et al. 2013). Moreover, Lohmander et al. (2006) has found for all ages that there were similar THA indications in the Nordic countries. A more stringent diagnostic approach might have taken place in some countries and over the years, explaining the development in this indication.

Systematic inflammatory disease as indication has declined over the years. The decline in SID as indication for surgery was expected since powerful disease-modifying drugs (DMDs) have been on the market for more than 20 years. The decline in THA patients with SID might even continue during the next decade.

The differences in SID as indication between the different countries may be reflecting that the incidence and prevalence of inflammatory arthropathies have been varying in different reports. Berntson et al. (2003) found an incidence of juvenile arthritis in the Nordic countries varying between 5/100,000 and 36/100,000 in different areas.

In accordance with Metcalfe et al. (2018), we did not find associations between indications for THA and implant survival. Hannouche et al. (2016) also found the same when he studied 91 patients, 113 hips, in patients younger than 20 years with THAs with ceramic-on-ceramic bearings. Several authors have found inferior implant survival in SIDs patients, especially in older series (Roach et al. 1984, Chmell et al. 1997, McCullough et al. 2006). One could expect SID patients to be less physically active than other young people and thus that the prosthesis would last longer. On the other hand, using DMDs may have helped patients to be physically more active over the years, hence increasing the risk of wear. We assume that our data on the youngest SID patients showed better survival because DMDs have had a favorable effect on morphological changes in the joint before surgery took place, therefore making the surgery less demanding. NARA data for THA patients in all ages have previously shown low revision rates in patients with pediatric hip disease. Engesaeter et al. (2012) found a 10-year survival of 94% after pediatric hip disease treated by THA for all ages. We could not find such a favorable trend for pediatric hip disease patients, which may be explained by different ages in the populations.

Unadjusted 5-year implant survival was 94% in our material; 10-year survival was 86% and 15-year survival 73%. This is a 5-year implant survival comparable to the 96% survival in the recent national register study by Metcalfe et al. (2018). Our result is also comparable to a recent publication from the University of Utah with 145 THAs in patients 30 years and younger included from 2000 to 2015 with a 10-year implant survival of 89% (Makarewich et al. 2018). Our implant survival is considerably better than results reported in a Norwegian study with surgery performed 1987–2010, which had a 10-year survival of 70% with endpoint any revision (Tsukanaka et al. 2016). The difference in survival might be explained by our more recent study period (1995–2016), which did not include inferior implants and bearing surfaces from the 1980s (Havelin et al. 2002).

During recent years there has been a trend in the literature towards using uncemented implants, which are thought to perform better in younger patients, but there are diverging results. In a systematic review Gee et al. (2013) found that among 450 primary THAs in patients aged less than 30 years uncemented stems did better than cemented. Schmitz et al. (2013), conversely, found a 10-year survival of 86% with 69 cemented THAs in patients younger than 30 years in a Dutch study. Wroblewski et al. (2010) reported a 65% implant survival at 19 years in 39 hips with cemented Charnley arthroplasties. Mean age at surgery was 18 years in his study. Data from the Australian Orthopaedic Association National Joint Replacement Registry collected from September 1999 through December 2012 of 297 THAs in patients younger than 21 years unveiled a 5-year revision rate of 4.5%. Many of the implants were resurfacings (Sedrakyan et al. 2014). We found that cemented and uncemented implants had similar survivorship (Table 4). Hybrid fixation had an adjusted HR of revision of 2.5 but, due to the relatively small numbers of THAs in each fixation group, the confidence intervals are wide and tend to overlap. Larger numbers would have given us more precise estimates and hence a clearer picture of survivorship for the different fixations (Figure 4).

Figure 4.Kaplan–Meier unadjusted survival curves with confidence intervals (shaded areas) for different fixation methods with uncemented fixation as reference. (a) Uncemented versus cemented; (b) uncemented versus hybrid; (c) uncemented versus reverse hybrid. For adjusted survival see Table 4. 31 resurfacing arthroplasties were not included.

**Figure:**
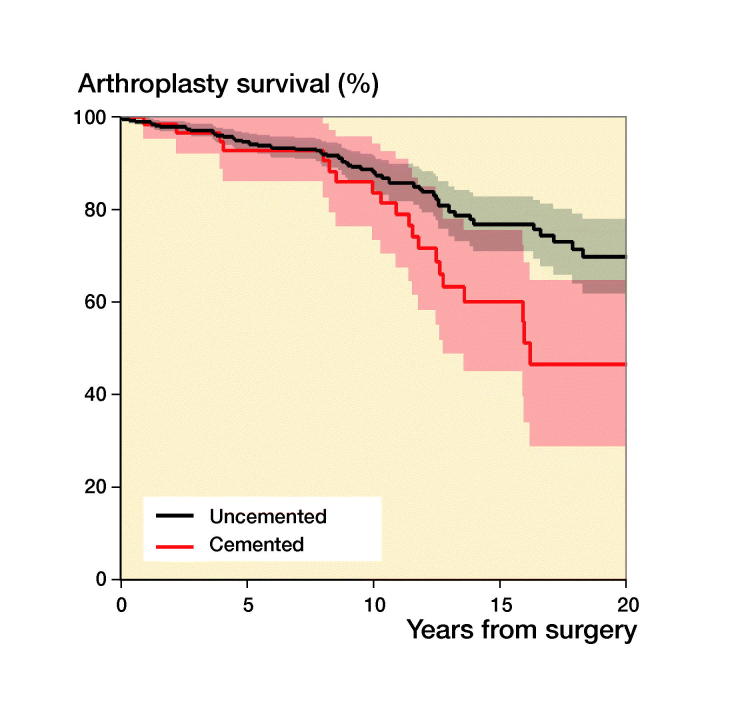

**Figure:**
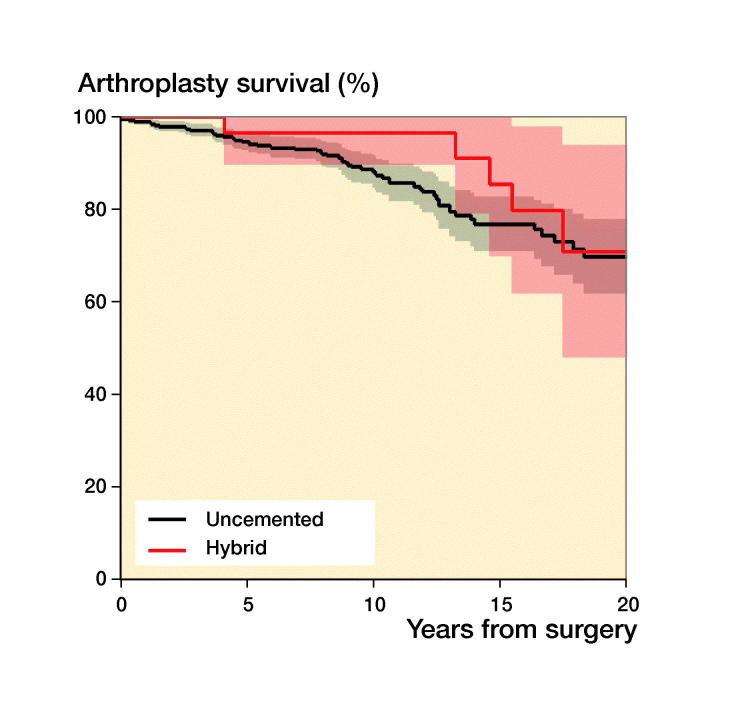

**Figure:**
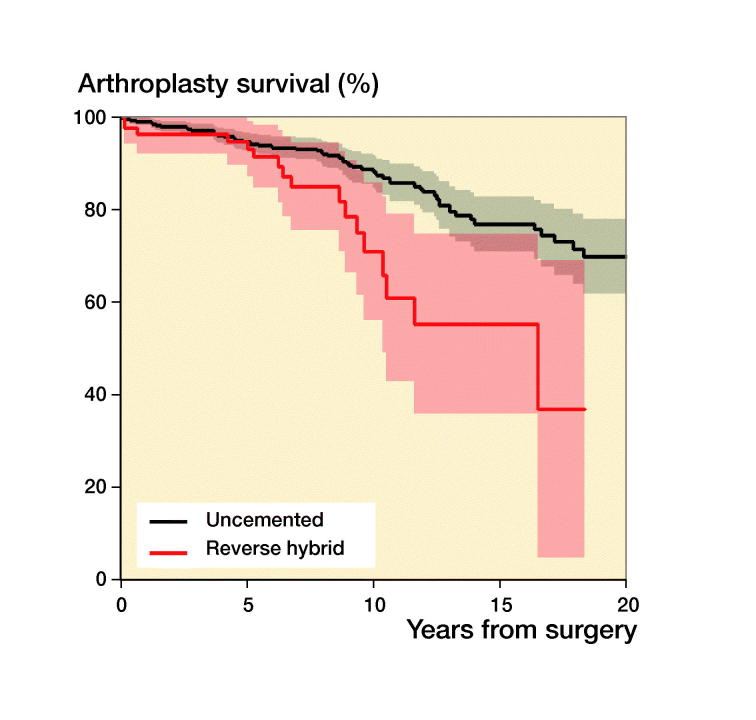

We found similar adjusted HRs for revision between the 3 time periods analyzed (Table 4) and for the period 2012–2016 compared with 1995–2011 (data not shown). The lack of a favorable trend over time might be due to the fact that in 2015 there were only 27 hips at risk (Figure 2) and only 5 revisions were recorded in the period 2012–2016.

The main cause of revision in our study was aseptic loosening, which is in accordance with reports from earlier studies (Dudkiewicz et al. 2003, Pakos et al. 2014, Sedrakyan et al. 2014, Hannouche et al. 2016). There were only 9.3% dislocations as cause of revision, even though 46% of the patients were operated with head sizes smaller than 32 mm (Table 4) and 46% of them were combined with posterior approach. 37% had head size smaller than 28 mm combined with a posterior approach (data not shown in table). A speculation might be that younger patients have stronger muscles around the hip preventing dislocations to a greater extent than older patients. The number of deep infections was low, but is under-reported in registers. There might have been soft tissue debridements that were not reported. Older patients often have comorbidities that make them more frail and susceptible to infection.

Although our dataset is the largest to date, it should be interpreted with caution. The NARA minimal dataset contained only information common to all the registers in Denmark, Finland, Norway, and Sweden. A weakness in the study is that data on articulations, head sizes, surgical approaches, and revision procedures were not recorded during the entire study period for all countries. Complementing and harmonizing the Nordic register data is an ongoing process.

All countries used many different acetabular and femoral components. The combinations of components made the material as a whole highly heterogeneous. The wide diversity of component designs jeopardizes a more detailed analysis of cemented and cementless components.

In summary, analyzing data from the NARA dataset on 881 total hip replacements on patients 21 years or younger there was a decline in systemic inflammatory disease as indication for THA, and the overall survival at 10 years was 86% with reverse hybrid fixation showing less favorable survival. Survival for cemented and uncemented implants was the same adjusted for sex, indication, head size, and time period for index surgery.

**Table:** Patients at risk in Figures 2–4

Years from surgery:	0	2	4	6	8	10	12	14	16	18	20
Figure 2:	881	738	597	474	357	272	196	132	99	60	27
Figure 3:	710	584	462	361	269	201	145	101	72	43	19
Figure 4: Uncemented	659	540	422	330	243	185	127	85	70	45	24
Cemented	62	56	50	45	40	36	28	19	11	3	1
Hybrid	36	30	28	25	22	20	19	16	10	6	1
Reverse hybrid	78	71	63	45	29	14	9	6	4	2	0
